# Supplementary material for: Structural order in plasmonic superlattices
Source: Nat Commun. 2020 Jul 30;11:3821. doi: 10.1038/s41467-020-17632-4 (PMC7393164; doi:10.1038/s41467-020-17632-4)
Supplement: Supplementary file 1 — Supplementary Information [file 41467_2020_17632_MOESM1_ESM.pdf]

Supplementary Information

for

Structural order in plasmonic superlattices

Schulz et al.

TEM Characterization

Supplementary Fig. 1: Freestanding monolayer films on a bare TEM grid without amorphous carbon coating.

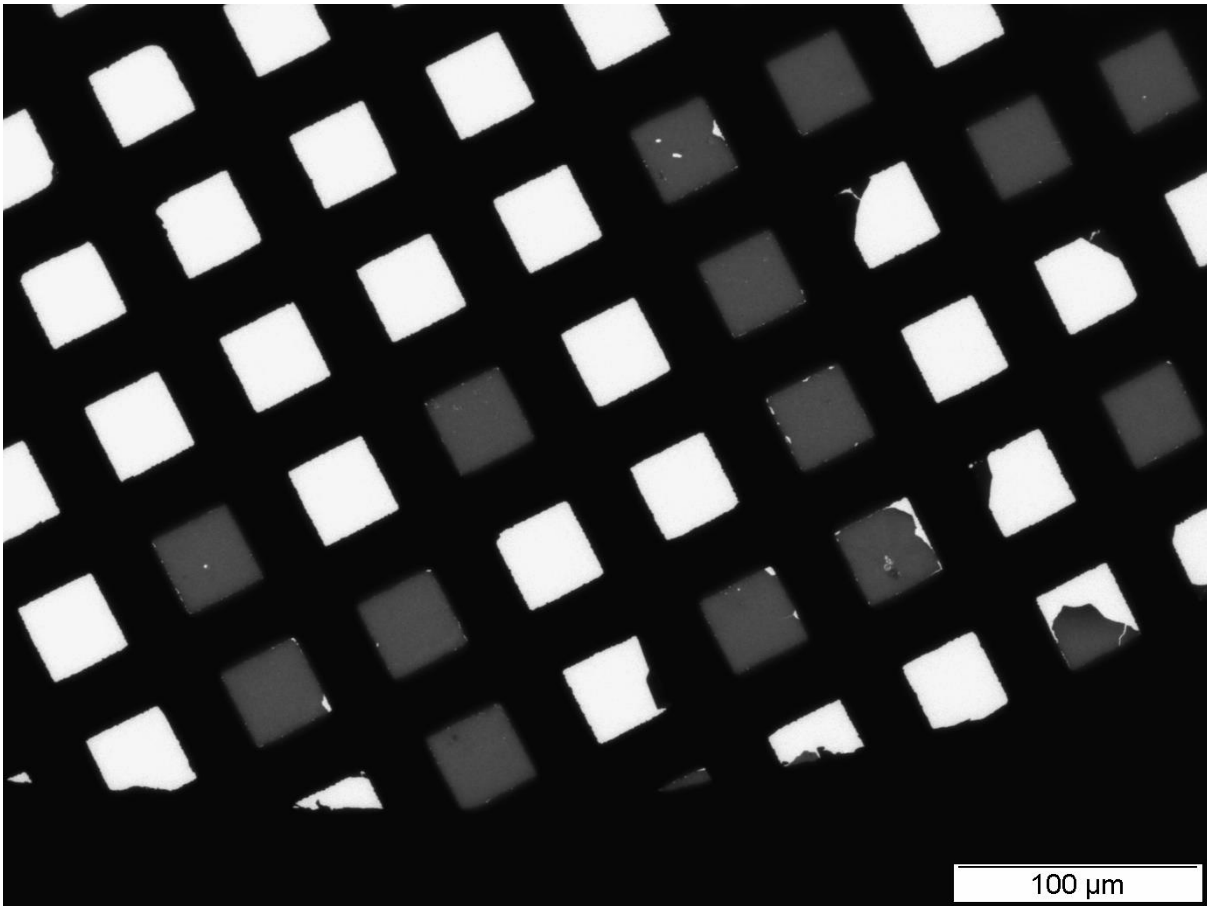

Nominal mesh dimensions: $(38.5\ \mu m)^2$ mesh, 25 µm bridge width.

Supplementary Fig. 2: AuNP25@PSSH2k.

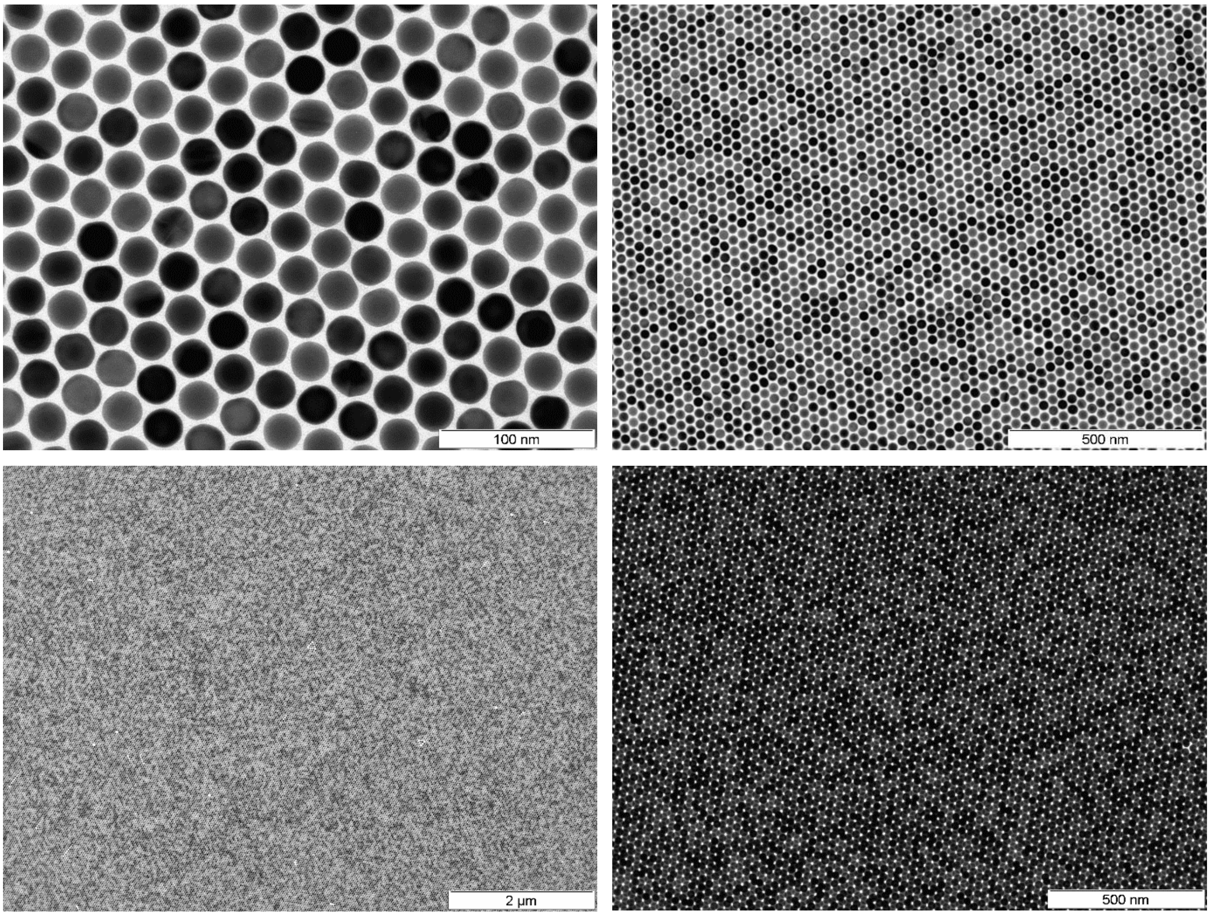

Monolayer regions at different magnifications and bilayer region (bottom right).

Supplementary Fig. 3: AuNP25@PSSH5k.

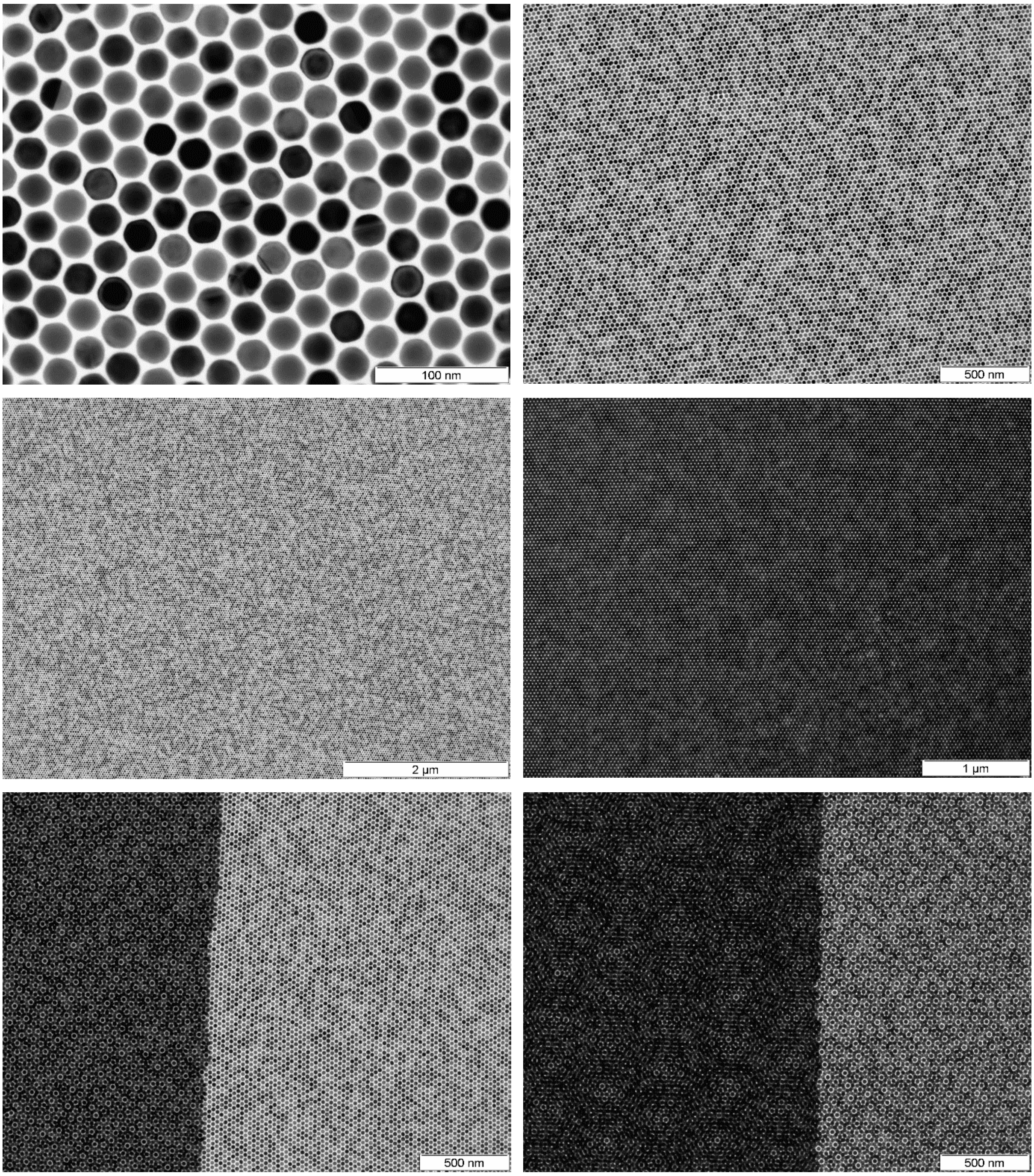

Monolayer region at different magnifications (top row and middle row, left), bilayer region (middle right), mono- and bilayer region with second layer overlapping slightly twisted resulting in Moiré-pattern (bottom left). Bi- and trilayer region with Moiré-pattern (bottom right).

Supplementary Fig. 4: AuNP25@PSSH12k.

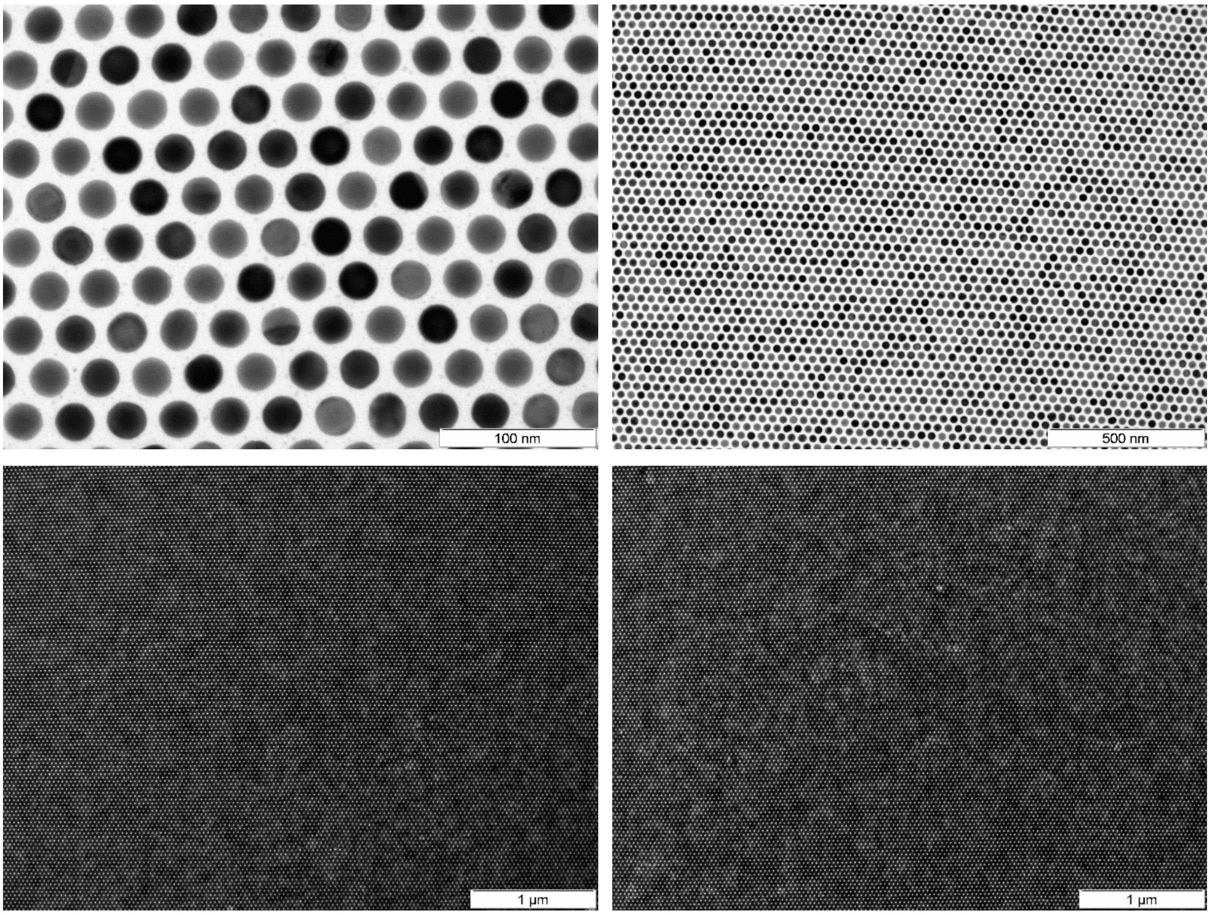

Monolayer region at different magnifications (top row), bilayer region (bottom left) and trilayer region (bottom right).

Supplementary Fig. 5: AuNP35@PSSH5k.

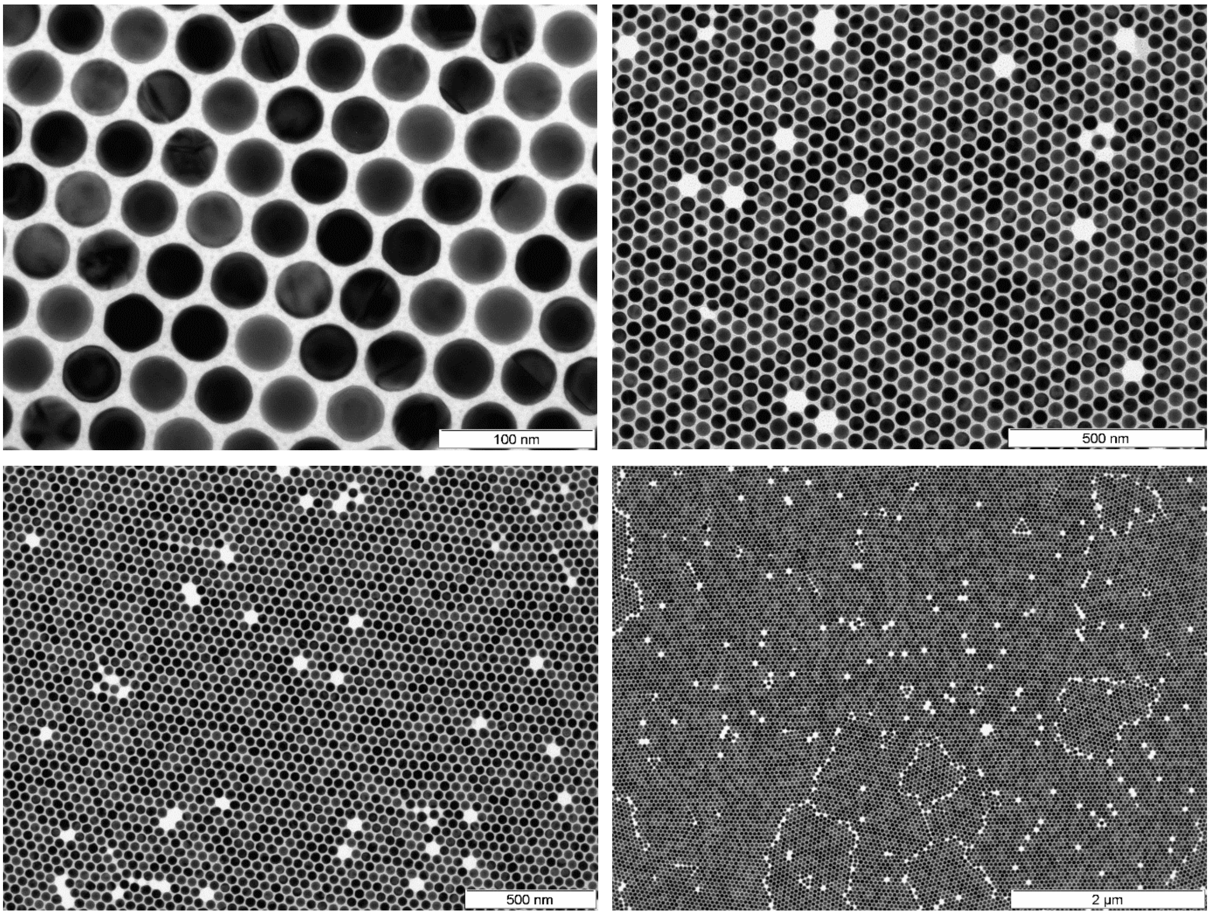

Monolayer regions at different magnifications. In this sample more defects formed.

Supplementary Fig. 6: AuNP35@PSSH12k.

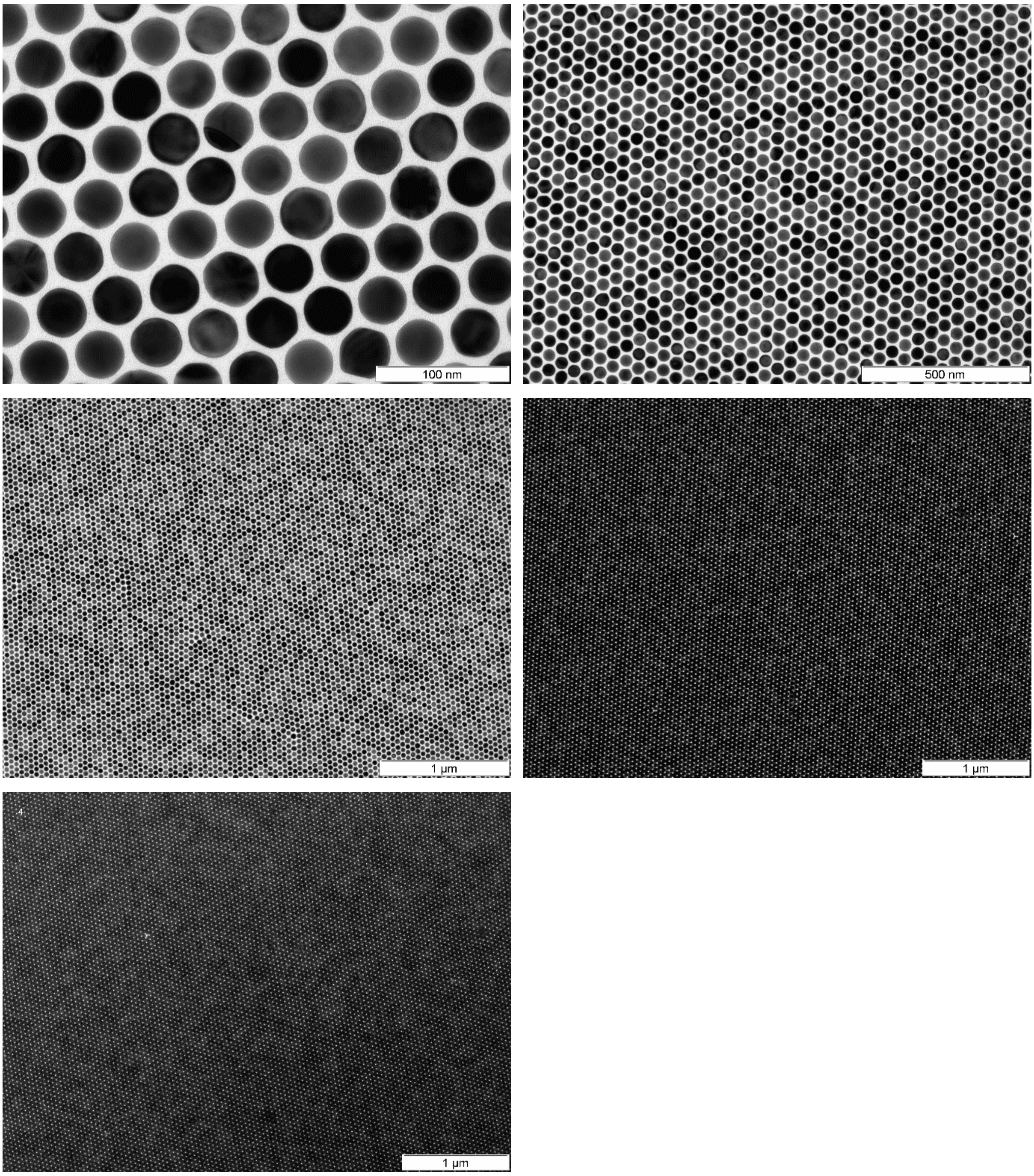

Monolayer regions at different magnifications (top row and middle left), bilayer region (middle right) and tetralayer region (bottom left).

Supplementary Fig. 7: AuNP50@PSSH5k.

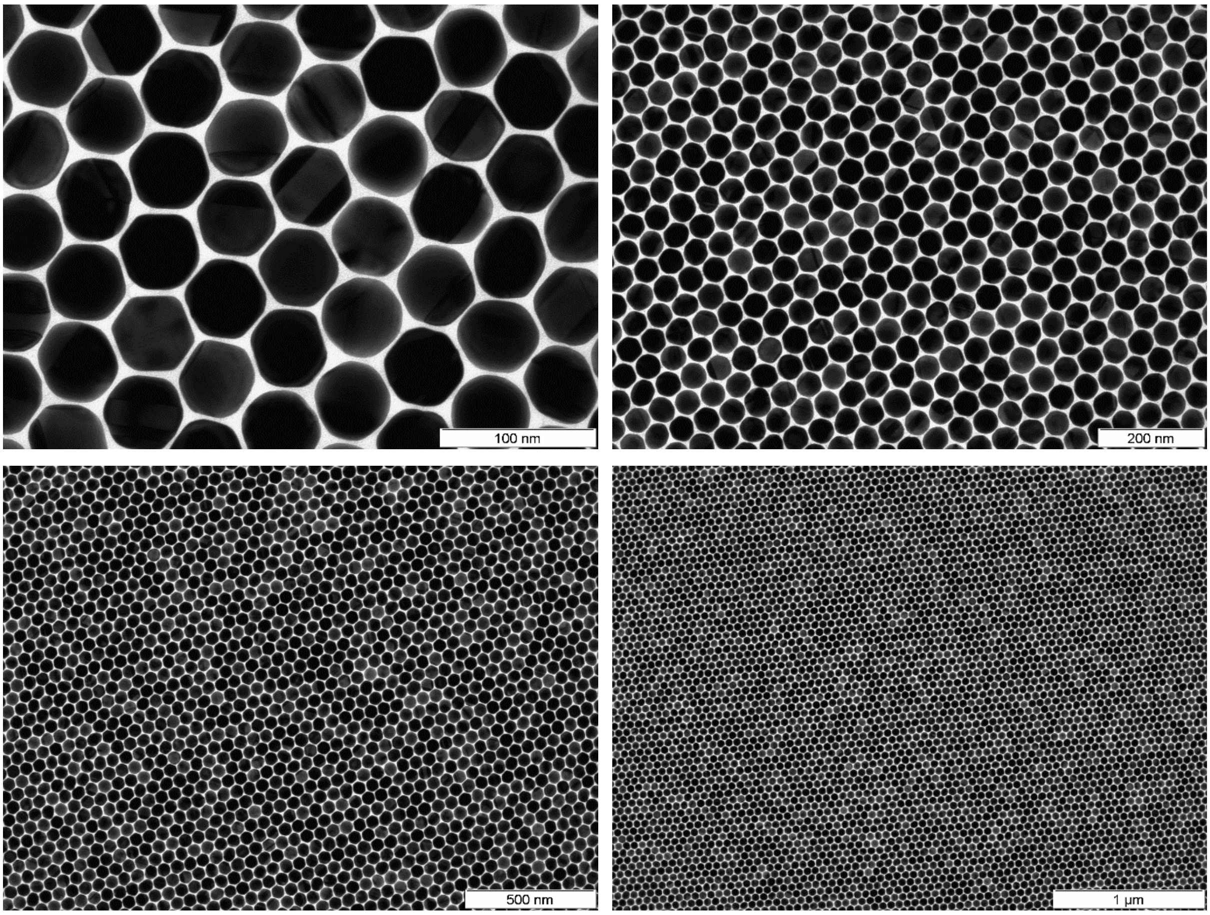

Monolayer regions at different magnifications.

Supplementary Fig. 8: AuNP50@PSSH12k.

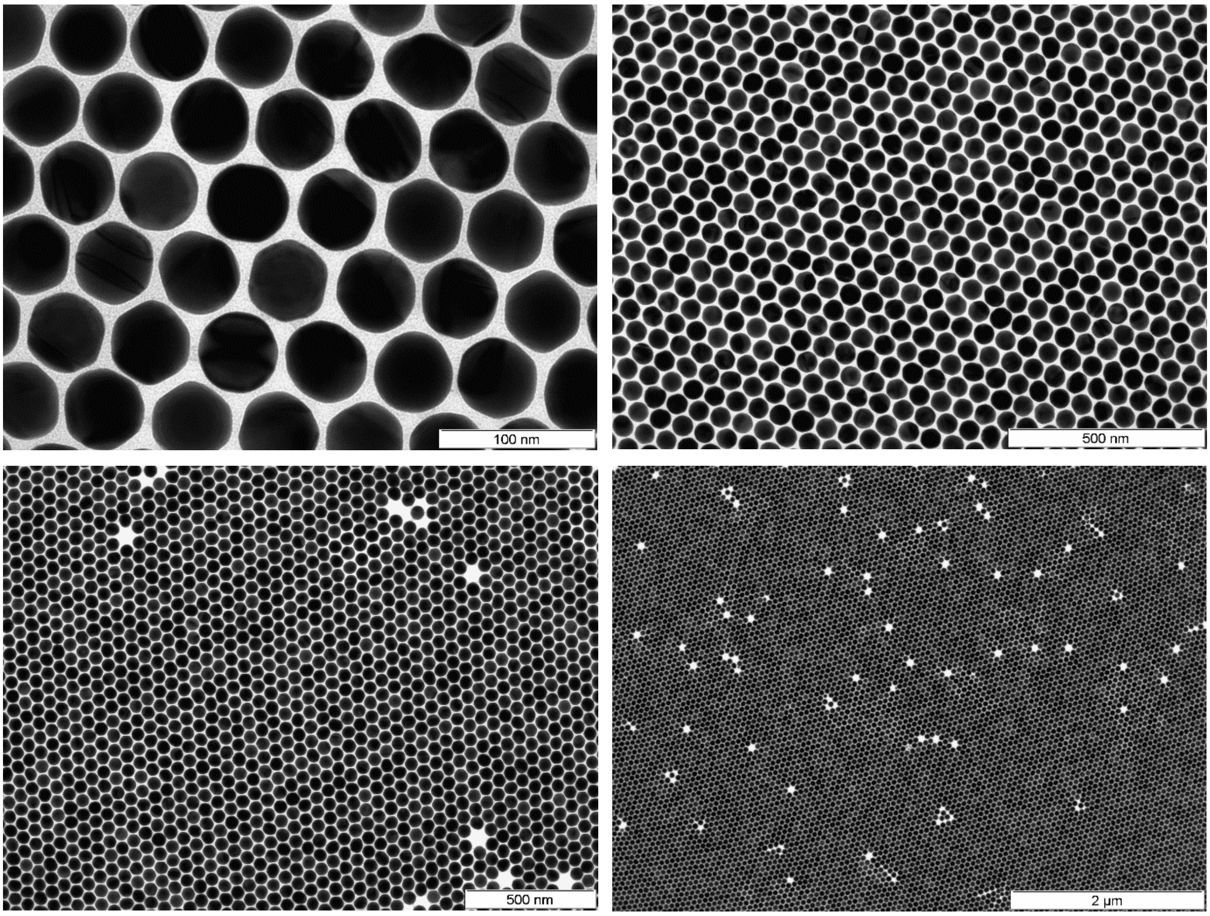

Monolayer regions at different magnifications.

Supplementary Fig. 9: AuNP60@PSSH2k.

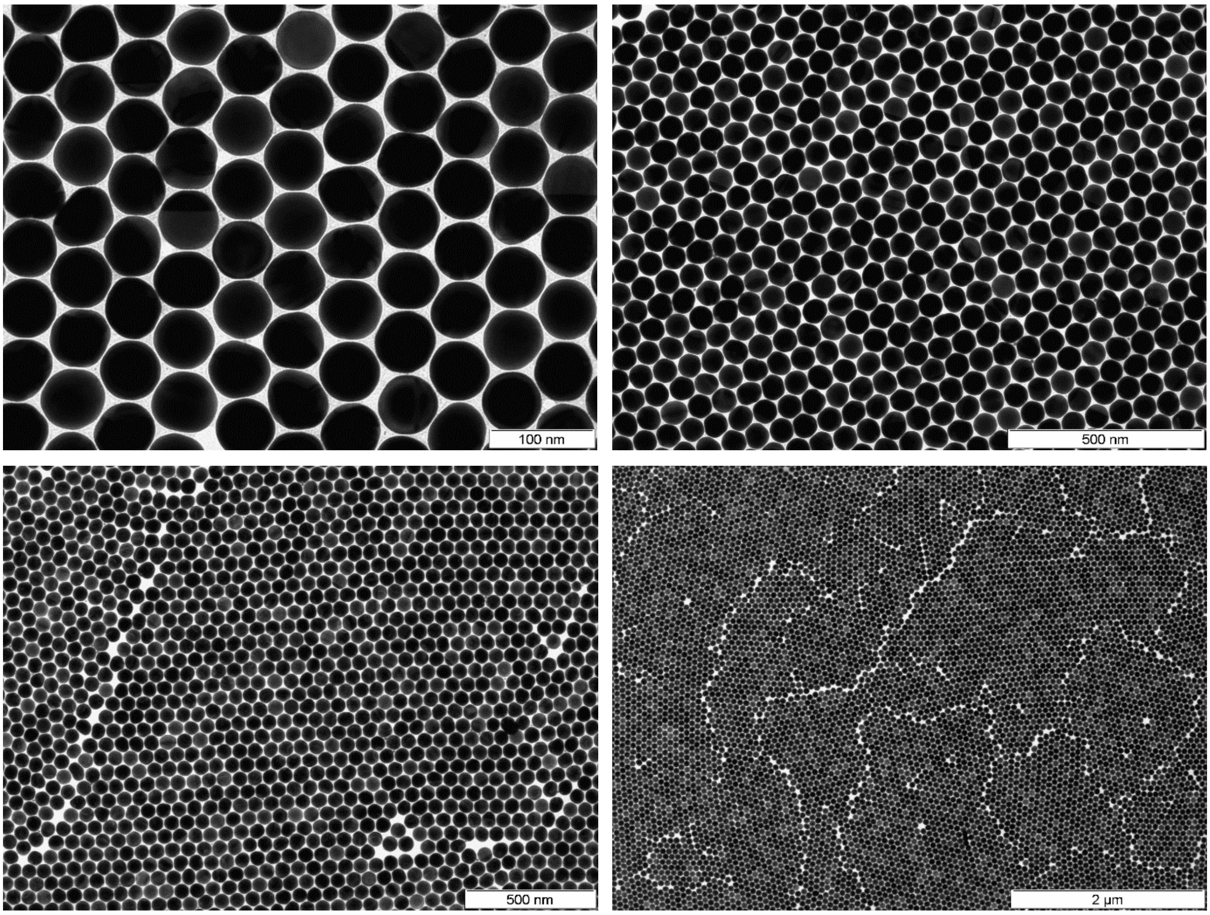

Monolayer regions with cracks and defects at different magnifications.

Supplementary Fig. 10: AuNP60@PSSH5k.

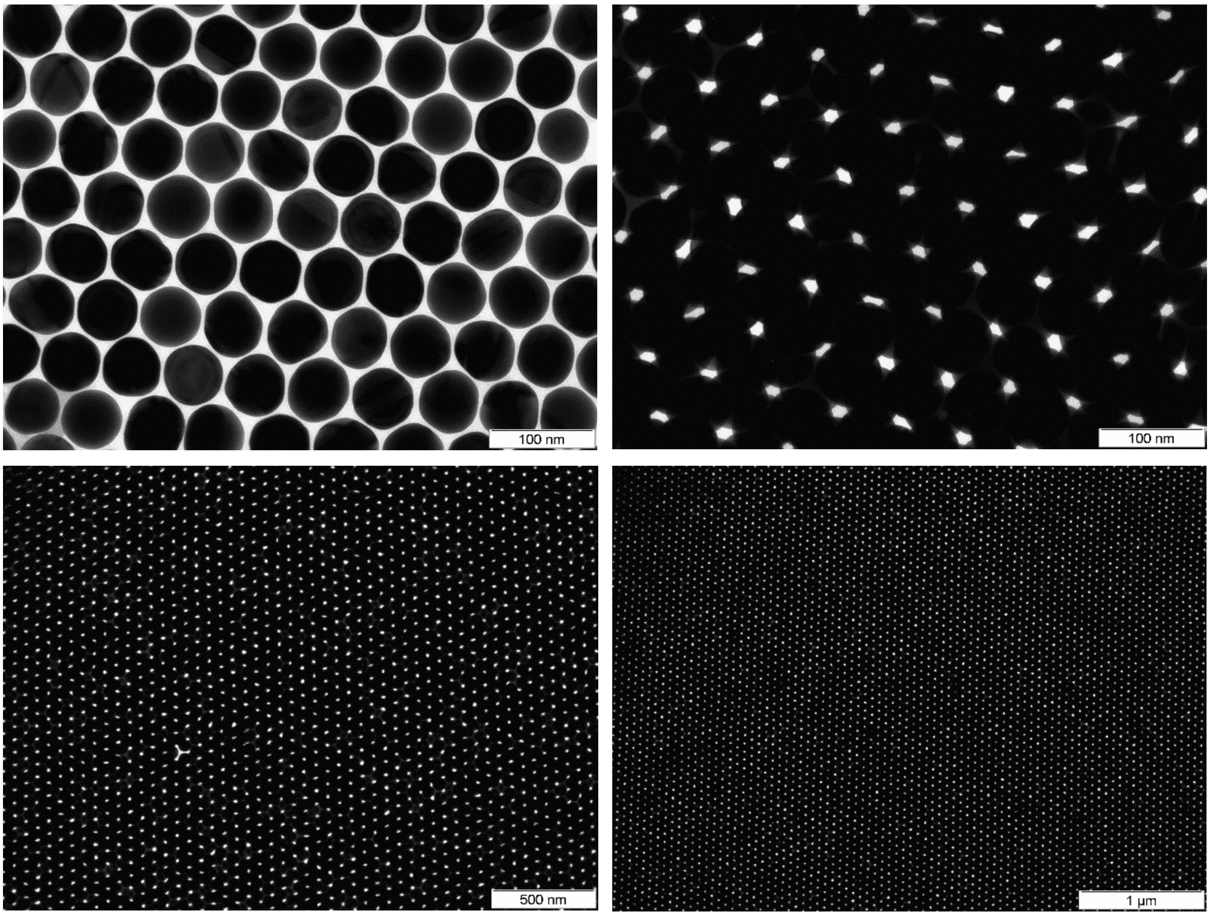

Monolayer region (top left) and bilayer regions (top right and bottom row). In this sample no extended monolayers were obtained but bi- and multilayers.

Supplementary Fig. 11: AuNP60@PSSH12k.

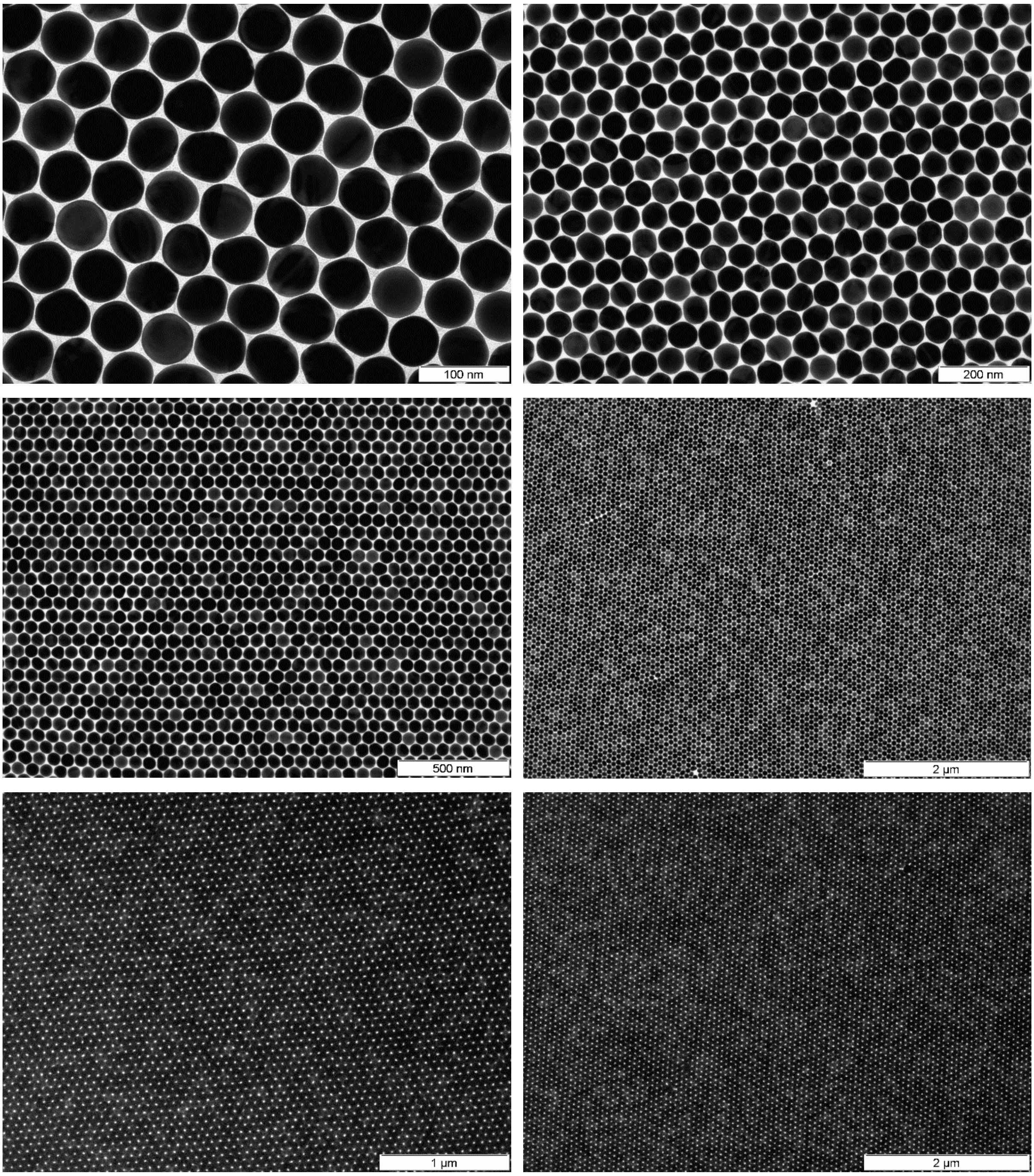

Monolayer regions (top and middle row) and bilayers (bottom row) at different magnifications.

SEM Characterization

Supplementary Fig. 12: Exemplary SEM measurements of crystalline mono- and multilayer structures (AuNP60@PSSH12k).

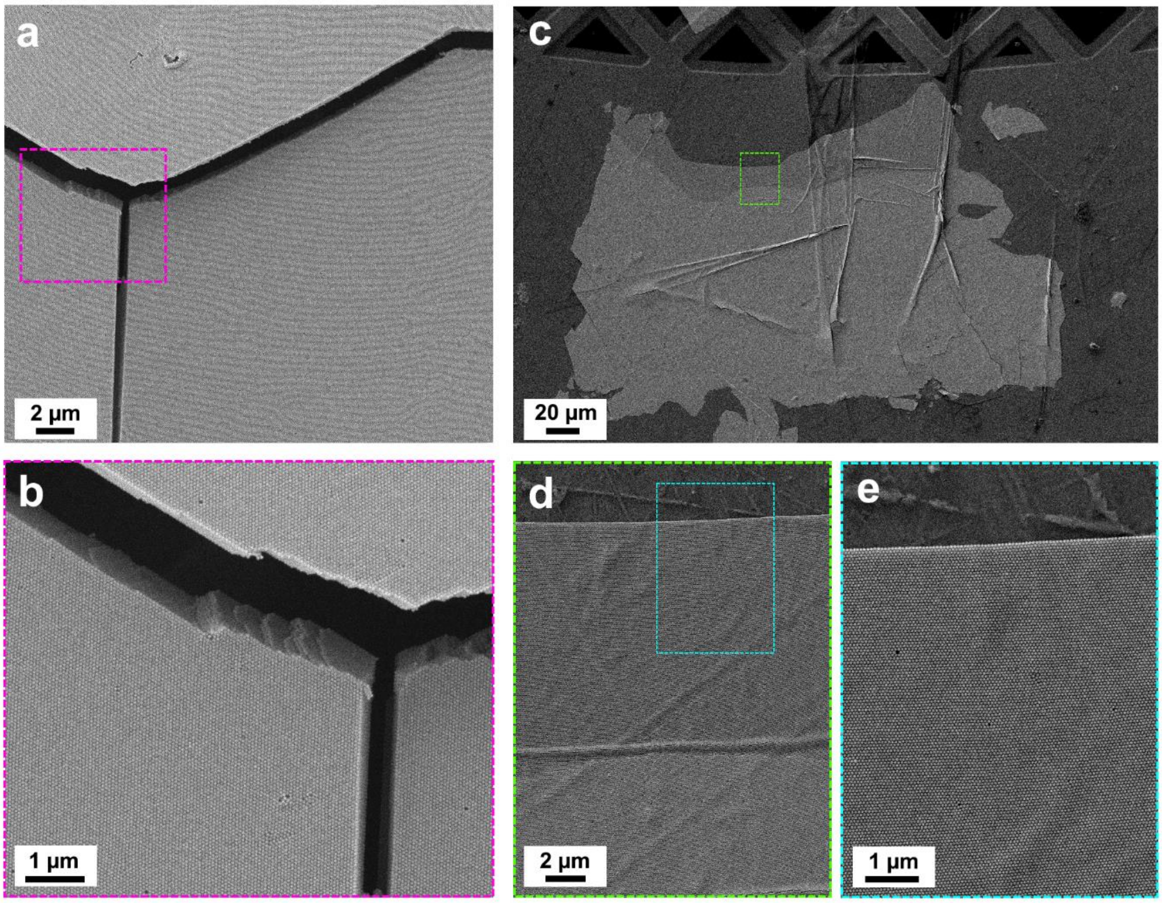

a Crystalline multilayer area. **b** Boxed area in **a** at larger magnification. **c** Bilayer superlattice fragment on the edge of a TEM grid. Cracks and wrinkles presumably result from the film transfer but maybe also from the structure of the substrate. The green boxed part in **c** is shown in **d** and the blue boxed part in **d** in **e**, identifying the structure as a bilayer with a smaller monolayer part.

Interparticle distances (gaps)

Supplementary Fig. 13: Correlation of interparticle distance (gap) and AuNP diameter.

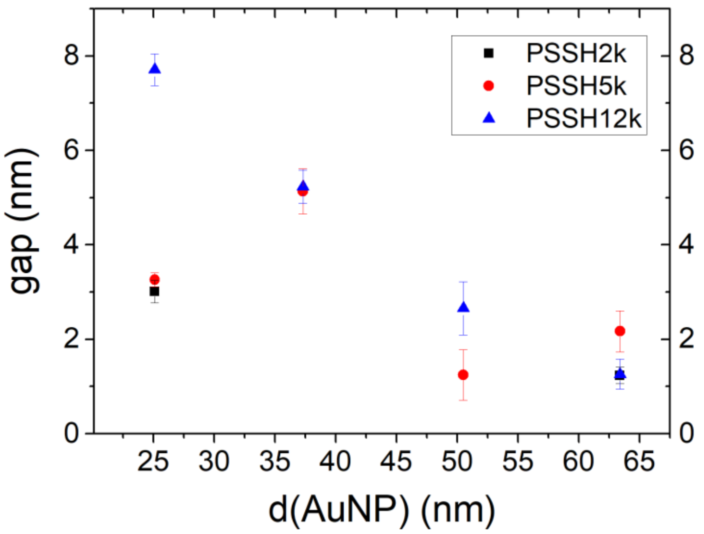

Optical microscopy of AuNP superlattices

Supplementary Fig. 14: Transmittance optical microscopy of AuNP25@PSSH2k.

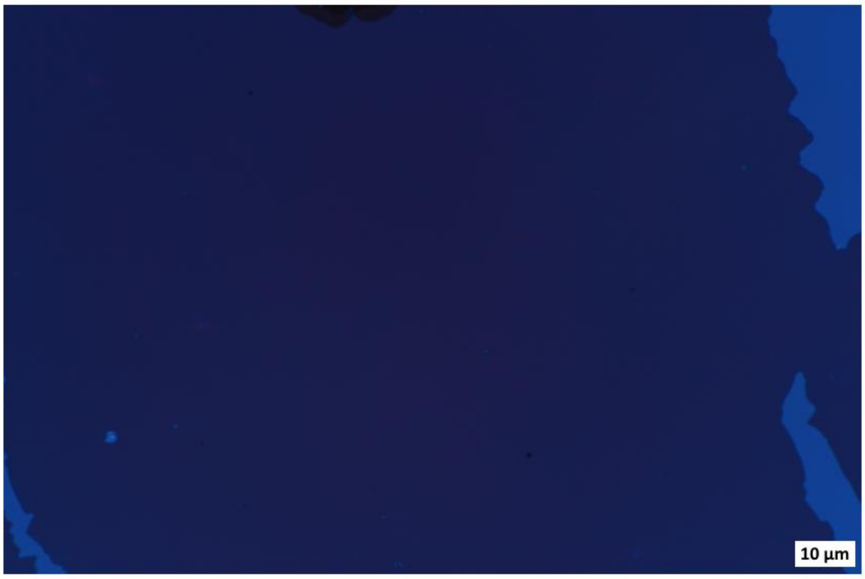

The large dark blue area is a ~ 100 µm x 100 µm trilayer formed from AuNP25@PSSH2k. The image was acquired using a white light source. The bilayer regions can be clearly discerned by their weaker contrast.

Surface-enhanced Raman scattering of an AuNP superlattice trilayer

Supplementary Fig. 15: SERS properties of an AuNP50@PSSH5k trilayer.

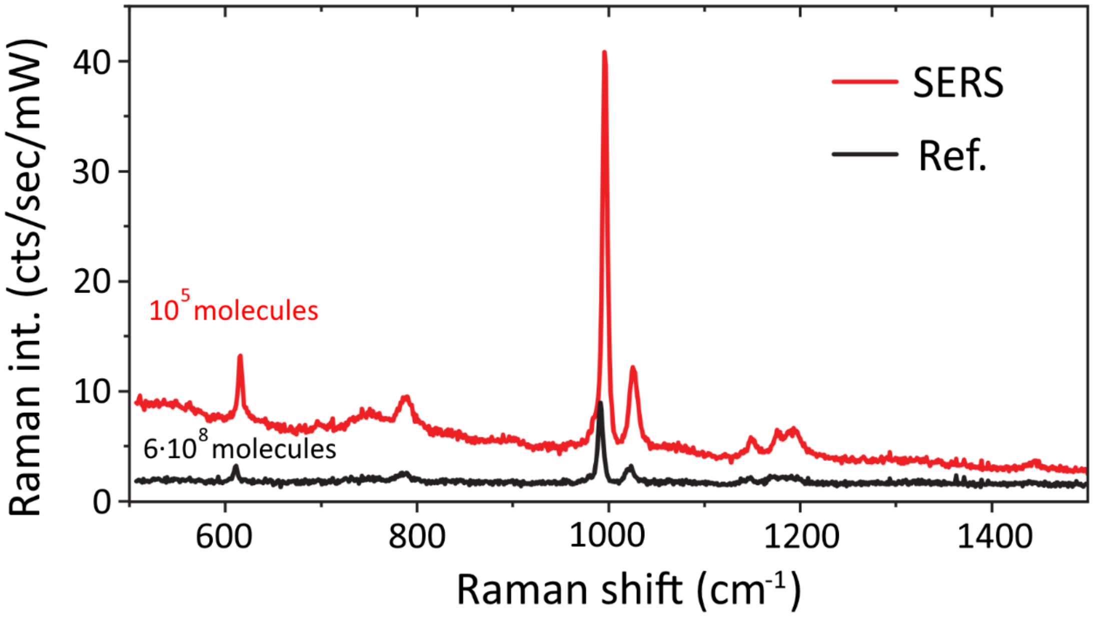

The SERS spectrum of the sample is dominated by the aromatic breathing modes of the polystyrene-ligands at 1000 cm^{-1}. Comparison with a reference Raman spectrum (Ref.) of the same ligand without AuNP yields an average enhancement factor of roughly $3 \cdot 10^4$ per molecule (detailed calculation provided below).

Supplementary Note 1. Surface-enhanced Raman spectroscopy (SERS). The SERS measurements were conducted with a Horiba XploRA single-grating confocal Raman spectrometer, equipped with a grating having 1200 grooves per mm. The sample was illuminated through a 100x objective with 0.9NA (Olympus MPlan N) with linearly polarized light with an excitation wavelength of 785 nm and laser power of 560 µW. A 50 µm pinhole and linear polarizer was inserted in the detection pathway. The SERS spectrum was measured at the same sample position were the spectra in Figure 3a in the main text were recorded. The sample was moved with a piezo stage and the position identified by optical reflectance microscopy. The reference spectrum was recorded with the same set up and settings on a polystyrene (PSSH5K) powder.

The SERS enhancement factor was estimated as follows:

Number of molecules that contribute to the SERS signal: The density of polystyrene-thiol molecules that cover the gold surface was assumed to be 1/nm^2 based on literature values.[1]

From FDTD simulations we estimated the size of one hotspot as 50 nm^2. As each hot spot contains molecules from two gold surfaces, one hot spot contains approximately 100 molecules. The laser spot size was approximately one μm^2. From the gold nanoparticle diameters (50.5 nm), interparticle gap sizes (1.3 nm) and layer number (3) we estimated 1200 hot spots inside the laser spot (because of the excitation with linearly polarized light we have one hotspot per AuNP). From this, we estimated that $\approx 10^5$ molecules are probed in the SERS measurement.

Number of molecules that contribute to the reference signal: For the reference measurement we used a powder of polystyrene molecules (PSSH5K) and focused/collected the light with the same microscope objective as for the SERS measurement. We estimated the extension of the laser spot in z direction as 5 μm which gives a volume of the 3D laser spot of 5 μm^3. The density of the polystyrene molecules is 1.05 g/cm^3 and molar density is $M_n = 5300$ g/mol which gives a number of $6 \cdot 10^8$ molecules inside the laser spot.

The SERS intensity of the aromatic breathing mode at 1000 cm^{-1} was by a factor 5 stronger than the intensity in the reference measurement (Supplementary Fig. 15). When accounting for the different number of molecules that were probed in the two measurements, we obtain a SERS enhancement factor of $5 \cdot 6 \cdot 10^8 / 10^5 \approx 3 \cdot 10^4$.

References

1. Ye, X. *et al.* Structural diversity in binary superlattices self-assembled from polymer-grafted nanocrystals. *Nat. Commun.* **6,** 10052; 10.1038/ncomms10052 (2015).
